# Double Agents in the War on Cancer: Leukocytes Govern Ovarian Cancer Progression

**DOI:** 10.18632/oncotarget.478

**Published:** 2012-04-15

**Authors:** Uciane K. Scarlett, Jose R. Conejo-Garcia

**Affiliations:** The Wistar Institute,Philadelphia, PA; The Wistar Institute,Philadelphia, PA

Although epithelial ovarian cancer is responsible for the deaths of >15,000 Americans per year, it remains a relatively under-researched disease. Emerging results suggest that only a small fraction of ovarian cancers (designated type I) persist as restrained but noticeable masses for extended periods [1]. In contrast, it has been proposed that the ovarian carcinomas that are responsible for 90% of deaths (designated type II) [1] could evolve aggressively without a localized macroscopic precursor lesion. To elucidate the unknown dynamics of ovarian cancer initiation and malignant progression, we generated a model of ovarian carcinoma that develops in healthy mice without oncogenic mutations during embryonic development, as happens in adult human tumors [2]. As independent studies have confirmed that in the ovarian carcinoma microenvironment T cells (and so far only they) can *spontaneously* exert clinically relevant pressure against tumor progression [3], we also aimed to understand the role of the immune system in this experimental model. For that purpose, we developed insidiously progressing, spontaneously antigenic, oncogen-driven tumors in immunocompetent adult mice. We therefore expressly avoided the use of artificial epitopes that do not reflect the mild nature of the tumor antigen recognition system.

Our results demonstrate that, after primordial tumor lesions are established, measurable spontaneous immune responses against cancerous cells control tumor growth for a relatively prolonged equilibrium phase. In fact, simply ablating p53 in the ovaries of adult mice did not result in tumor development in multiple experiments, suggesting that a previously healthy individual needs to accumulate enough mutations to develop a tumor faster than the immune system can eliminate it. Although future experiments need to substantiate this hypothesis, our data clearly show that even if aggressive tumors can be established, tumor microenvironmental leukocytes restrain them for a long latency period, during which tumors remain indolent (microscopic). However, once tumors become macroscopically noticeable, progression is extremely rapid. Our work therefore supports that some tumors evolve very aggressively, which has implications for redefining the goal of early detection strategies. Specifically, ovarian cancers (and likely other aggressive carcinomas) will need to be detected as microscopic lesions; otherwise, they may evolve too rapidly for successful treatment.

Expectedly, we found that tumor-specific T cell responses are initiated by dendritic cells (DCs). Immune control during the equilibrium phase is dependent on CD8 T cells, because their temporary depletion dramatically accelerates tumor progression. More interestingly, the key to the initiation of aggressive malignant expansion is in the phenotypic and numerical changes taking place in microenvironmental DCs. Thus, exponential growth starts when phenotypically distinct DCs outnumber T cells in the tumor microenvironment (TME). We defined these cells as DCs because they express determinants such as CD11c, DEC205, CD86 and MHC-II. They also effectively process and present antigens in the right milieu [4, 5]. However, in advanced tumors, these leukocytes abrogate the activity of anti-tumor T cells. In humans, categorizing myeloid leukocytes in the ovarian carcinoma microenvironment is complex due to inter- and intra-tumor heterogeneity and pathological mobilization of immature precursors. Nevertheless, we also found that in at least a third of freshly dissociated human solid tumors analyzed, the most frequent leukocyte subset expressed DC makers but not CD11b or CD14 (determinants of monocytes/macrophages). Our emphasis on defining this population goes beyond semantics, because we found that these DCs are as good as eliciting T cell responses as they are at abrogating them. Therefore, interventions that re-programmed their capacity to boost protective immunity *in vivo*, could achieve the dual goal of eliminating an immunosuppressive component and activating the immune system. Our recent work has demonstrated the feasibility of this approach through administration of functional miRNA mimetics to tumor-bearing hosts [6].

Overall, our results underscore the importance of the immune system's([a-z]) modulation of tumor progression, and have major physiopathological and therapeutic implications:

Firstly, our data indicate that the balance between immune protection and tolerance drives cancer progression, without any direct intervention on tumor cells. Correspondingly, depleting DCs at early stages in tumor-bearing mice accelerates tumor progression, but at later stages prevents exponential growth, again allowing our immune system to recognize the tumors. As iterations of “surgery+chemotherapy” have resulted in modest improvements in the prognosis of the most aggressive cancers in the last 40 years, targeting immunosuppression could offer significant hope. This is better illustrated by the recent success of CTLA4 and PD1 blockade [7].

Secondly, we found that advanced tumors remain immunogenic. Although, overall, our evidence supports the “*cancer immunoediting*” hypothesis (the current framework in tumor immunology[8]), these results introduce a tweak; namely, that the crucial event driving aggressive malignant expansion is leukocyte-mediated immunosuppression, rather than loss of recognizable tumor antigens.

Thirdly, the success of promising immunotherapies such as T cell transfer or improved vaccination may require concurrent targeting of immunosuppressive mechanisms for implementation against aggressive epithelial cancers.

Fourthly, although T cells are still able to exert some spontaneous anti-tumor immune pressure, their activity is decreased at advanced stages, even when they are separated from immunosuppressive leukocytes.

In conclusion, our work expands our understanding of tumor progression and provides further mechanistic rationale to develop novel interventions targeting immunosuppression. Future studies should unveil other unrecognized aspects of the contribution of the immune system to cancer prevention and progression.
